# Supplementary material for: A capacitive laser-induced graphene based aptasensor for SARS-CoV-2 detection in human saliva
Source: PLoS One. 2023 Aug 17;18(8):e0290256. doi: 10.1371/journal.pone.0290256 (PMC10434860; doi:10.1371/journal.pone.0290256)
Supplement: S1 File — (DOCX) [file pone.0290256.s001.docx]

**A capacitive laser-induced graphene based aptasensor for SARS-CoV-2 detection in human saliva**

Geisianny Moreira ^1,2,3^, Hanyu Qian ^4^, Shoumen Palit Austin Datta ^5,6^, Nikolay Bliznyuk ^4^, Jeremiah Carpenter ^7,8^, Delphine Dean ^7,8^, Eric McLamore ^1,2,3*^, Diana Vanegas ^1,3,9*^

^1^ Environmental Engineering and Earth Sciences, Clemson University, Clemson, SC, USA

^2^ Department of Agricultural Sciences, Clemson University, Clemson, SC, USA

^3^ Global Alliance for Rapid Diagnostics, Michigan State University, East Lancing, MI, USA

^4^ Department of Agricultural and Biological Engineering, University of Florida, Gainesville, FL, USA

^5^ Department of Mechanical Engineering, MIT Auto-ID Labs, Massachusetts Institute of Technology, Cambridge, MA, USA

^6^ Department of Anesthesiology, Medical Device (MDPnP) Interoperability and Cybersecurity Labs, Biomedical Engineering Program, Massachusetts General Hospital, Harvard Medical School, Cambridge, MA, USA

^7^ Center for Innovative Medical Devices and Sensors (REDDI Lab), Clemson University, Clemson, SC, USA

^8^ Department of Bioengineering, Clemson University, Clemson, SC, USA

^9^ Interdisciplinary Group for Biotechnology Innovation and Ecosocial Change -BioNovo, Universidad del Valle, Cali, Colombia

*Corresponding authors

E-mail: dvanega@clemson.edu (DV), emclamo@clemson.edu (EM)

**Supporting Information**

1. ***In silico* modeling of aptamer structure**
   1. *Literature review*

Three aptamers were identified in the literature related to SARS-CoV-2 detection that meets the requirements for selection (**S1 Table**).

**S1 Table.** *In silico* modeling for three aptamers targeting SARS-CoV-2 selected from the literature. *In silico* modeling was performed using RNAfold (Gruber et al. 2008), using the minimum free energy (MFE), partition function algorithm, and DNA parameters (Matthews, 2004) with a folding temperature of 37 °C.

| **Aptamer ID** | **Molecular**  **target** | **Length**  **[nt]** | **GC content**  **[%]** | **Minimum free energy (DG) [kcal/mol]** | **Melting**  **temp. ™**  **[°C]** | **Molecular weight [kDa]** | **Reference for discovery of aptamer** |
| --- | --- | --- | --- | --- | --- | --- | --- |
| CoV2-RBD-1C | Receptor-Biding Domain RBD | 61 | 44.9 | -12.40 | 73.80 | 18.8 | Song et al. 2020. |
| SNAP1 | N-terminal Domain NTD | 60 | 48.5 | -13.70 | 75.10 | 18.3 | Kacherovsky et al. 2021. |
| MSA52 | S1 subunit | 89 | 53.9 | -6.70 | 79.1 | 27.9 | Zhang et al. 2022. |

- 1. *In silico modeling for MSA52 aptamer by mFold and RNAfold web servers*

The MSA52 aptamer (Zhang et al. 2022) was chosen for its ability to bind SARS-CoV-2 wildtype and its variants of concern. MSA52 aptamer binds the Spike protein of either SARS-CoV-2 wildtype and its variants of concern (Alpha, Beta, Gamma, Epsilon, Delta, Omicron BA.1) with K_D_ values ranging from 2 to 10 nM (Zhang et al. 2022). We have included a T-tail and a biotin tag to the SELEX native sequence to facilitate the linker of aptamer to LIG electrode surface by streptavidin-biotin coupling method. Aptamer MSA52 was selected for detailed analysis (based on minimum free energy, more stable when low; number of variants tested, tested viral particle). Aptamer sequences (native and scrambled) are shown below:

MSA52

[Bio-TEG] TTTTTTTTTT

TTACGTCAAGGTGTCACTCCGTAGGGTTTGGCTCCGGGCCTGGCGTCGGTCGTCTCTCGCGAAGCATCTCTTTGGCGTG

T-Scrambled MSA52

[Bio-TEG5] TTTTTTTTTT TTACGTTTTTTTTTTTCTCCGTAGGGTTTGGCTCCGGGCCTGGCGTCGGTCGTCTCTCGCGAAGCATCTCTTTGGCGTG

- - 1. *mFold characterization of MSA52 aptamer*

**S2 Table** shows the main results for MSA52 modeling using mFold web server regarding the structural predicted as a high probability.

**S2 Table**. MSA52 Aptamer modeling by mFold. Modeling was performed for native and T-scrambled aptamer sequences without linker. Secondary structure modeling conditions: 1M Na+ and 1M Mg++, folding temperature at 37 °C.

| Aptamer | Stem/loop ratio (slr)  (nt_s_/nt_l_) | GC content  (%) | Minimum free energy (ΔG) (kcal/mol) | Molecular weight (kDa) | Extinction coefficient  (M^-1^ cm^-1^) |
| --- | --- | --- | --- | --- | --- |
| Native MSA52 | 1.48 | 54 | -11.97 | 27.9 | 796.10 |
| T-scrambled MSA52 | 0.61 | 48 | -10.66 | 27.4 | 777.2 |

**S1 Fig** shown one native and one T-scrambled structural predicted as a high probability by the model for MSA52 aptamer. The mFold predicted one structure for native MSA52 with a ΔG of -11.97 kcal/mol, and three structures for T-scrambled MSA52, with a ΔG of -10.66 kcal/mol (structure 1), -10.22 kcal/mol (structure 2), and -10.21 kcal/mol (structure 3).

**S1 Fig.** **Predicted secondary structure of aptamer MSA52 by mFold web server.**

(A) Native sequence from Zhang et al. (2022). (B) T-scrambled sequence (position 6 to 16 modified with T substitution).

- - 1. *RNAfold characterization of MSA52 aptamer*

**S3 Table** shows the main results for MSA52 modeling using RNAfold web server regarding the structural predicted as a high probability.

**S3 Table**. MSA52 Aptamer modeling by RNAfold. Modeling was performed for native and T-scrambled aptamer sequences without linker. Secondary structure modeling conditions: minimum free energy (MFE), partition function algorithm, and DNA parameters (Matthews, 2004) with a folding temperature of 37 °C.

| Aptamer | Stem/loop ratio (slr)  (nt_s_/nt_l_) | GC content  (%) | Minimum free energy (DG) (kcal/mol) | Molecular weight (kDa) | Extinction coefficient  (M^-1^ cm^-1^) |
| --- | --- | --- | --- | --- | --- |
| Native MSA52 | 1.26 | 54 | -7.00 | 18.9 | 796.10 |
| T-scrambled MSA52 | 0.68 | 48 | -6.50 | 18.4 | 777.2 |

**S2 Fig** shows one native and one T-scrambled structural predicted as a high probability by the model for MSA52 aptamer. One structure was predicted for both native and T-scrambled sequences by RNAfold. Mountain plots generated for each predicted structure are also shown in **S2 Fig**.

**S2 Fig**. **Predicted secondary structure and mountain plots of aptamer MSA52 by RNAfold (Vienna) web server.**

(A) Native sequence from Zhang et al. (2022). (B) T-scrambled sequence (position 6 to 16 modified with T substitution).

1. **OPC as a technique to guarantee sample integrity**

**S4 Table** shows the pH of several substances used to determine interference in sensing testing.

**S4 Table. pH and temperature of samples tested by OCP technique.**

| Sample type | pH sample at 10% | T (℃) |
| --- | --- | --- |
| Human saliva | 8.5 | 24 |
| Pooled saliva | 8.5 | 24 |
| Artificial saliva | 8.6 | 24 |
| Tap water | 8.7 | 24 |
| Orange juice | 5.4 | 23 |
| Gatorade | 7.2 | 23 |
| Coffee | 8 | 23 |
| Synthetic urine | 8.8 | 24 |
| NaCl/NaHCO_3_ | - | 24 |

1. **Aptasensor baseline characterization**

**S3 Fig** shows a representative voltammogram for triplicates selected based on overlapping oxidation/reduction peaks and the similarity between voltammogram shapes.

**S3 Fig.** **Triplicates selection. Representative cyclic voltammogram of the LIG-nPt electrode replicates *vs.* Ag/AgCl (3M KCl) in 100 mM KCl and 2.5 mM K_3_[Fe(CN)_6_]/K_4_[Fe(CN)_6_] at scan rates of 200 mV s^−1^.**

**S4 Fig** shows complementary aptasensor baseline characterization during biofunctionalization to select a suitable response variable.

**S4 Fig.** **Electrochemical baseline characterization.**

(A) Representative Bode Impedance plot (log f *vs.* Z) in EIS non-Faradaic mode (physiological solution (NaCl/NaHCO_3_) supplemented with 10% (v/v) pooled saliva). (B) Representative Bode Impedance plot (log f *vs.* Z’) in EIS non-Faradaic mode (physiological solution (NaCl/NaHCO_3_) supplemented with 10% (v/v) pooled saliva). (C) Representative Bode Capacitance plot (log f *vs.* Cs) in EIS non-Faradaic mode (physiological solution (NaCl/NaHCO_3_) supplemented with 10% (v/v) pooled saliva). (D) Representative Bode Capacitance plot (log f *vs.* C’) in EIS non-Faradaic mode (physiological solution (NaCl/NaHCO_3_) supplemented with 10% (v/v) pooled saliva).

1. **Confocal images of sensor biofunctionalization**

To corroborate the presence and distribution of biotin-tagged aptamer onto the LIG-nPt electrode surface, confocal microscopy images were taken on a Leica SPE confocal (Leica Microsystems, Wetzlar, Germany) at the Clemson Light Imaging Facility (Clemson Division of Research, Clemson University, Clemson SC, US). Biotinylated aptamer tagged with Fluorescein at 3’extremity was drop-cast onto LIG-nPt electrodes surface, as described in the methods section. **S5 Fig** shows confocal imaging of LIG before (LIG-bare) and after loading the aptamer.

**S5 Fig.** **Confocal imaging of sensor biofunctionalization.**

(A) Confocal imaging of bare LIG. (B) Confocal imaging of fluorescein-tagged aptamer showing the aptamer distribution on the LIG-nPt electrode surface. Imaging was performed using the Leica SPE confocal. Scale bars: 1000 µm.

1. **Non-linear model for calibration curve**

**S5 Table** shows the non-linear model (MnMolecular1) fitted to obtain the calibration curve and used to estimated LOD.

**S5 Table.** MnMolecular1 non-linear model for aptasensor calibration curve. The data analysis was performed in the OriginLab software.

| **Model** | MnMolecular1 |
| --- | --- |
| **Equation** | y = A1 - A2*exp(-k*x) |
| **Plot** | ΔC'' |
| **A1** | 0.16124 ± 0.01927 |
| **A2** | 0.10608 ± 0.02446 |
| **k** | 0.00107 ± 6.01206E-4 |
| **Reduced Chi-Sqr** | 1.17557 |
| **R-Square (COD)** | 0.95041 |
| **Adj. R-Square** | 0.85123 |
